# Changes in network connectivity during motor imagery and execution

**DOI:** 10.1371/journal.pone.0190715

**Published:** 2018-01-11

**Authors:** Yun Kwan Kim, Eunhee Park, Ahee Lee, Chang-Hwan Im, Yun-Hee Kim

**Affiliations:** 1 Sungkyunkwan University School of Cognitive Science, Seoul, Republic of Korea; 2 Department of Physical and Rehabilitation Medicine, Kyungpook National University Chilgok Hospital, Daegu, Republic of Korea; 3 Department of Health Sciences and Technology, Samsung Advanced Institute for Health Science and Technology, Sungkyunkwan University, Seoul, Republic of Korea; 4 Department of Biomedical Engineering, Hanyang University, Seoul, Republic of Korea; 5 Department of Physical and Rehabilitation Medicine, Center for Prevention and Rehabilitation, Heart Vascular Stroke Institute, Samsung Medical Center, Sungkyunkwan University School of Medicine, Seoul, Republic of Korea; University of Minnesota, UNITED STATES

## Abstract

**Background:**

Recent studies of functional or effective connectivity in the brain have reported that motor-related brain regions were activated during motor execution and motor imagery, but the relationship between motor and cognitive areas has not yet been completely understood. The objectives of our study were to analyze the effective connectivity between motor and cognitive networks in order to define network dynamics during motor execution and motor imagery in healthy individuals. Second, we analyzed the differences in effective connectivity between correct and incorrect responses during motor execution and imagery using dynamic causal modeling (DCM) of electroencephalography (EEG) data.

**Method:**

Twenty healthy subjects performed a sequence of finger tapping trials using either motor execution or motor imagery, and the performances were recorded. Changes in effective connectivity between the primary motor cortex (M1), supplementary motor area (SMA), premotor cortex (PMC), and dorsolateral prefrontal cortex (DLPFC) were estimated using dynamic causal modeling. Bayesian model averaging with family-level inference and fixed-effects analysis was applied to determine the most likely connectivity model for these regions.

**Results:**

Motor execution and imagery showed inputs to distinct brain regions, the premotor cortex and the supplementary motor area, respectively. During motor execution, the coupling strength of a feedforward network from the DLPFC to the PMC was greater than that during motor imagery. During motor imagery, the coupling strengths of a feedforward network from the PMC to the SMA and of a feedback network from M1 to the PMC were higher than that during motor execution. In imagined movement, although there were connectivity differences between correct and incorrect task responses, each motor imagery task that included correct and incorrect responses showed similar network connectivity characteristics. Correct motor imagery responses showed connectivity from the PMC to the DLPFC, while the incorrect responses had characteristic connectivity from the SMA to the DLPFC.

**Conclusions:**

These findings provide an understanding of effective connectivity between motor and cognitive areas during motor execution and imagery as well as the basis for future connectivity studies for patients with stroke.

## Introduction

Motor imagery (MI) constitutes a mentally rehearsed task in which a given movement is imagined without the occurrence of actual movement [1]. Although an MI task is only performed mentally, imagining a movement recruits areas of the brain that are activated when actual movement is performed [2]. Motor areas of the cerebral cortex involved in motor execution (ME) consist of the primary motor cortex (M1) and several premotor areas, including the supplementary motor area (SMA), pre-supplementary motor area (pre-SMA), and ventral and dorsal parts of the premotor cortex (PMC). Similar to ME, motor areas of the cerebral cortex involved in MI include M1, SMA, pre-SMA, and ventral and dorsal parts of the PMC, although activation of M1 during MI is weaker compared to that during ME [3–5]. They also include areas related to action planning, such as the dorsolateral prefrontal cortex (DLPFC), inferior frontal cortex (IFC), and posterior parietal cortex (PPC) [6–9]. M1 is typically associated with ME. Moreover, the amount of M1 activation during MI reflects the level exhibited during execution [10]. Locations of SMA activity for ME and MI overlap only partially [11, 12]. The SMA is reportedly the most consistently active area and plays an important role in MI tasks as well as in high-level motor control [13–17]. The SMA is also involved in the programming of movements [18, 19]. The SMA plays a role in internally generated movements or actions that require sequences of movements [20]. The PMC exhibits overlapping activity during ME and MI and plays a role in the planning and preparation phases before simulation [13, 21]. The prefrontal and frontal cortices play a significant role in cognitive and motor events that instantiate action planning and programming [22]. Whether the prefrontal cortex is required for control of movement tasks guided by representations or internalized models of reality remains unclear [23].

Functional connectivity describes statistically temporal correlations between spatially remote brain areas during rest, but it does not provide any directional information or how these correlations are mediated. Functional connectivity is assessed by imaging during task-free, resting states; whereas the definition of effective connectivity is that one brain region exerts influence over another brain region by task. Effective connectivity is estimated from neuroimaging data collected during task performance [24,25]. It has been suggested that effective connectivity, perceived as experimental and time-dependent, needs the simplest schematic map that can reproduce the observed timing relationships between recorded network nodes [26]. When using DCM of functionally relevant signals, the locations of different configurations of sources and their connections can be tested to identify networks generated during task-related responses [27]. Therefore, DCM is currently used for the analysis of functional imaging, such as functional magnetic resonance imaging (fMRI), magnetoencephalography (MEG), and EEG [28, 29].

Previous studies have suggested that the prefrontal area is activated during both executed and imagined tasks [30]. Sequential organization of cortical neuronal events (action plans) was shown to be produced during MI. Although this sequential processing was planned simultaneously when performing actual motor control, the processing may consist of several separate parallel sub-processes [31]. Possibly, a similar neuronal substrate through the timing of the actual voluntary activity could affect temporal organization during MI. The timing of mentally rehearsed simulated movements is instantiated at a higher level than motor execution [23, 30, 32]. Another study used the Granger causality mapping (GCM) method to investigate effective connectivity in the brain during MI by selecting the SMA as the region of interest (ROI) [33]. The result showed that forward and backward effective connectivity was present between the SMA and three regions, including the bilateral dorsal premotor area (PMd), and the contralateral primary and secondary somatosensory cortices (S1). Based on the fMRI data sets obtained during ME and MI conditions, Kasess et al., [19] concluded that SMA suppression resulted in absence of M1 activation. This showed the SMA-suppressed movement preparation and performance by the motor system.

As mentioned, previous studies showed the prefrontal area was activated during performance of both ME and MI. We hypothesized that subjects’ cognition would show effects in the motor area of the brain including M1, PMC, and SMA during performance of the ME task, as the role of the PFC is action planning and programming [6, 23] which affects the ME and MI tasks. In addition, effective connectivity studies have not included the prefrontal area. Therefore, the objectives of this study were to analyze the effective connectivity between brain regions within motor and cognitive networks during ME and MI in healthy subjects. Moreover, the differences in effective connectivity between correct and incorrect responses during MI were analyzed using DCM on EEG data.

## Material and methods

### Participants

Twenty healthy subjects (mean ± standard deviation: 25.7±3.1, range 20–35 years, 10 females) participated in this study. The exclusion criteria were as follows: presence of any medical disorder, neurological disorder, orthopedic disorder, or substance abuse, history of taking psychoactive drugs or intracranial metal insertion. Subjects between 18 and 35 years of age were selected. During the experiment, subjects sat in a comfortable armchair watching a monitor from a distance of approximately 1 m. All subjects were right-handed, as determined by the Edinburgh Handedness Inventory [34]. Prior to the task, all participants were assessed using the Movement Imagery Questionnaire-Revised second version (MIQ-RS) [35], which consists of seven visual and seven kinesthetic items. This questionnaire uses a Likert scale, ranging from 1, “very difficult to see/feel,” to 7, “very easy to see/feel.” All participants provided written informed consent, and the study protocol was approved by the local ethics committee of Samsung Medical Center, Seoul, Korea.

### Motor task experiment

Presentation (Neurobehavioral Systems, Inc., Albany, CA, USA) was used for the motor task. The task consisted of a motor execution and a motor imagery sequence, which involved imagining the feeling of finger-tapping to mentally rehearse a finger-tapping sequence. Each participant performed the task with his or her dominant hand. EEG was recorded during the finger-tapping task. A motor task paradigm that provided evidence for task compliance by individual subjects was previously suggested and involved visual presentation of numbers to guide sequential finger-tapping execution and imagery. The motor task was divided into execution and imagery sessions. Subjects performed both execution and imagery sessions on the same day, the sequence of which was assigned randomly. In this study, we altered two aspects of this previous experimental design. First, instead of number stimuli, we used dot stimuli (range of dots) to guide the tapping sequence. Second, instead of a fixed quantity of stimuli in individual trials, we used a varied number of dot stimuli in each trial. We inferred that these modifications would deter subjects from employing alternative strategies, such as explicit counting, when performing the task and focus instead on the desired visuomotor strategy. We assumed that our subjects performed explicit imagery which could be imagined visually or kinesthetically. Before modifications in the motor task, alternative strategies such as explicit counting could interfere with motor imagery because the subjects might conflate visual information processing due to visual presentation of numbers to guide and recollect finger movements during the imagery task. Dot stimulation in each trial, however, relatively reduced visual information processing compared to when numbers were displayed.

**Fig 1:**
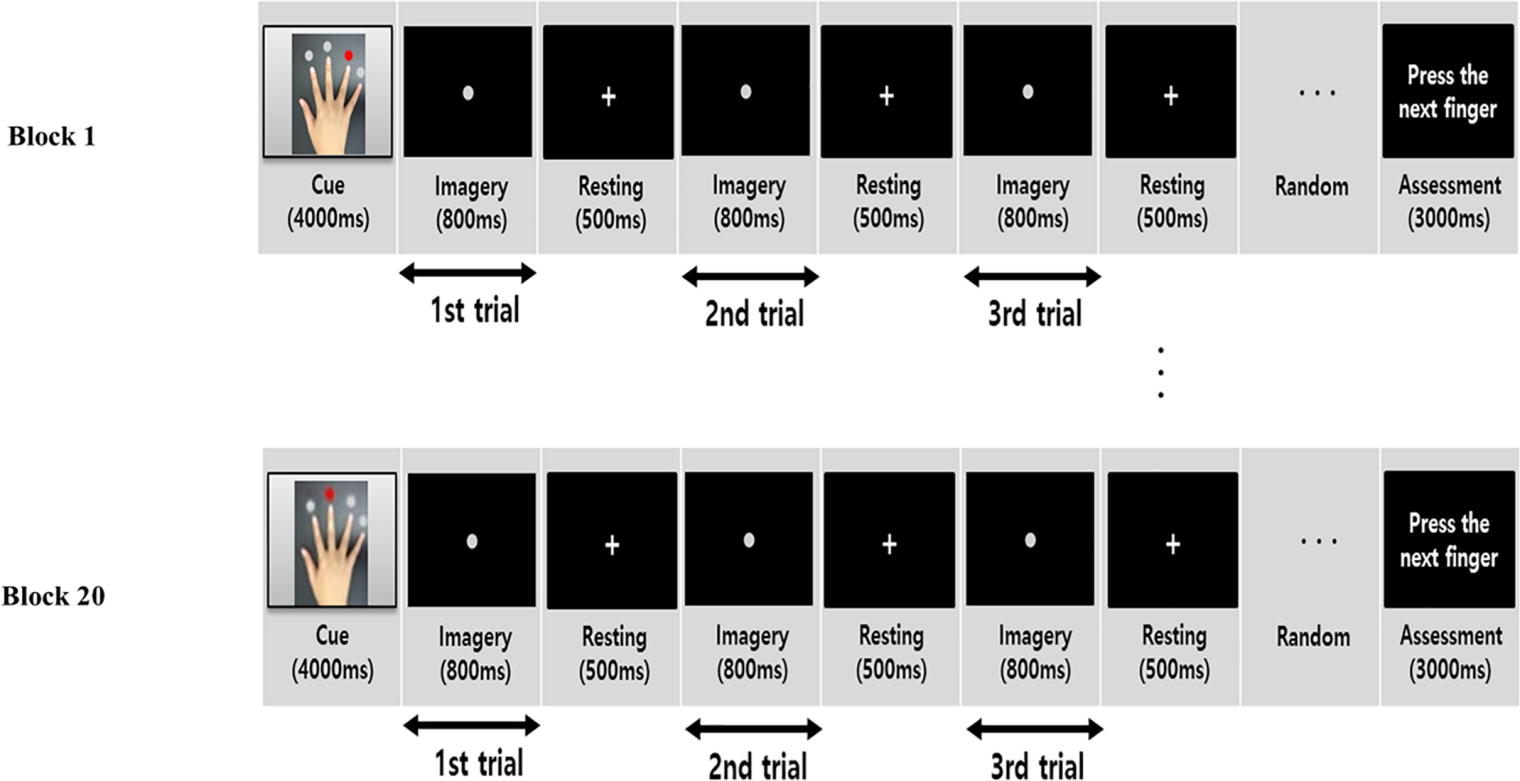
Motor task block components. The starting finger was indicated with a red dot. The experimental paradigm was divided into two sessions of motor execution and imagery. Each session was performed individually. A white dot was presented in the middle of the monitor every 1300 ms as a signal to progress to the next finger, both for motor execution and imagery. Each block was composed of at least three trials. Subjects pressed the appropriate button at the end of the task block as directed.

The experimental paradigm was designed to observe the motor task and objectively assess the performance of a task comprised of 20 trial-varied blocks. All blocks had at least three trials, although some blocks included five trials. A total of 20 blocks was defined as a task. In each block, a cue stimulus that specified the starting finger for tapping was presented for 4.0 sec, and then a series of at least three dot trials were presented at 1.3-sec intervals. Specifically, a dot appeared for 0.8 sec, and then a plus sign appeared for 0.5 sec. The subjects imagined or executed tapping of each finger when presented with only a dot, tapping in order from the radial to ulnar side and excluding the thumb. In the execution session, subjects tapped the indicated keyboard keys. In contrast, subjects only imagined the tapping movements during the imagery session. At the end of each block, the subjects were asked to physically press the next finger in the sequence in both execution and imagery tasks. Tapping with the correct or incorrect finger reflected the subject’s compliance with the ME and MI in each block (Fig 1). We recorded correct or incorrect responses based on the final finger movement when asked to physically tap a finger at the end of a block. The subject’s performance of the motor task during the entire paradigm was quantified as the percentage of the number of correct or incorrect finger-tapping responses among the 20 blocks.

### EEG recording and data processing

EEG data were collected using the NEURO PRAX® EEG system (NeuroConn, Ilmeanau, Germany) with 32 surface Ag/AgCl electrodes mounted on a recording cap (EASYCAP, Woerthesee-Etterschla, Germany) according to the international 10–20 system referenced to the right earlobe electrode.

The continuous EEG signals were processed with MATLAB (8.0.0.783, R2012b, MathWorks, Natick, MA, USA) and the EEGLAB toolbox (v11.0.54b http://sccn.ucsd.edu/eeglab/). SPM12b (v6225 http://www.fi.ion.uci.ac.uk/smp) was used for statistical analysis of preprocessed EEG data on a scalp-by-frequency basis.

After data were exported to a research computer, EEG signals were preprocessed with EEGLAB and SPM12b, an open source toolbox based on MATLAB [36]. For EEGLAB, the EEG signals were collected at a frequency of 4,000 Hz, down-sampled to 512 Hz, band-pass filtered at a range of 0.1–50 Hz, and epoched between -1000 and 4000 ms with respect to the onset of dot stimuli in each block. This study divided the frequency dimension into two non-overlapping frequency bands: a mu band of 8–12 Hz and a beta band of 13–20 Hz [37]. Eye-blink correction was conducted using independent components analysis via the EEGLAB toolbox for MATLAB [36]. The data epoch threshold was between -100 and 100 ㎶. In the time-frequency domain, epoched data length ranged from 0 sec before displaying the first dot to 0.8 sec after presenting the first dot. EEG signals were estimated as event-related spectral perturbations across average blocks of the C3 (contralateral side) and C4 (ipsilateral side) electrodes. A topographic map showed the distribution of the event-related spectral perturbation (ERSP) of all channels over each correct trial corresponding to dot appearance: first trial of 0.0–0.8 sec, second trial of 1.3–2.1 sec, and third trial of 2.6–3.4 sec.

For SPM12b, the data were epoched offline with a peri-stimulus window of -1000 to 4000 ms. The data were down-sampled to 512 Hz and band-pass filtered in the range of 0.1–50 Hz. The artifact threshold for eye movements or muscular activity was set between 0 and 100. We defined the lowest amplitude threshold for artifact rejection as 0.2 uV. We removed artifacts related to eye movement or muscular activity only in blocks, rather than rejecting each trial, then interpolation of bad channels was performed.

### 3D source reconstruction

For DCM using SPM, EEG signals were converted into voxel-based images by generating a brain map of the scalp images provided by SPM over each correct or incorrect block. EEG-based 2D brain-frequency scalp images were generated and the 2D brain-frequency scalp image was transformed to match brain maps provided by SPM. These converted dimensions of the interpolated brain image were used for statistical analysis. The 2D brain-frequency statistical image that was used to create the brain map of the scalp was represented by topological inference [38]. These processes constituted necessary steps to obtain an imaging reconstruction of EEG data. Because anatomical MRI data were not available for each subject, we used a template head model. The template head model has been used when individual head models were not acquired, and the template head model might be precise because EEG electrode positions were transformed to match the head model template, and SPM employed the template head model based on the MNI brain. The MNI brain images are a well-defined template and previous research has validated the match between the template head model corresponding to structural images that included 8196 vertices of cortical template mesh and EEG electrode positions. [38]. Although each individual subject’s head is quite different from the template A match was partly achieved because the 8,196 vertices of the template have been validated as dense. Source orientations were assumed to be normal with respect to the cortical mesh [38]. The actual lead field matrix was determined using forward computation, which computes the effect exerted on the sensors for each of the dipoles in the cortical mesh. The result is an N x M matrix, where N is the number of sensors, and M is the number of mesh vertices. Each column in this matrix produces a lead field corresponding to one mesh vertex. We computed Maxwell’s equations and assumptions regarding the physical properties of the head. These are known as “forward models”. When the head model was ready, it was displayed in the graphics window with the cortical mesh, and sensor locations were verified. The actual lead field matrix was computed at the beginning of the next step and saved. We used the boundary element method (BEM) model in order to more accurately model the electric field propagation through brain tissue; this model produces better results and has been shown to decrease localization error [38–42]. The localization accuracy of bioelectric source reconstruction is improved by the BEM method. The forward problem might be solved numerically through transformation to a boundary integral equation when approximating conductance within the head by a volume conductor consisting of compartments with constant and isotropic conductivities [43]. For reconstruction based on an empirical Bayesian approach to localize evoked responses, we chose the time window between -1000 ms and 4000 ms for inversion. This time window means that, although all subjects performed 20 blocks, the 20 blocks required different time intervals because each block was assigned trials randomly, although each block always included three trials. Therefore, the data from three trials represented one block. The time window between -1000 and 4000 ms also included the preparation and performance periods. The reason why we selected this time window was to show the difference between the execution and imagining states. We used DCM to analyze event-related potentials in order to produce a spatiotemporal model of the full data set.

### Regions of interest (ROI) for DCM

**Fig 2:**
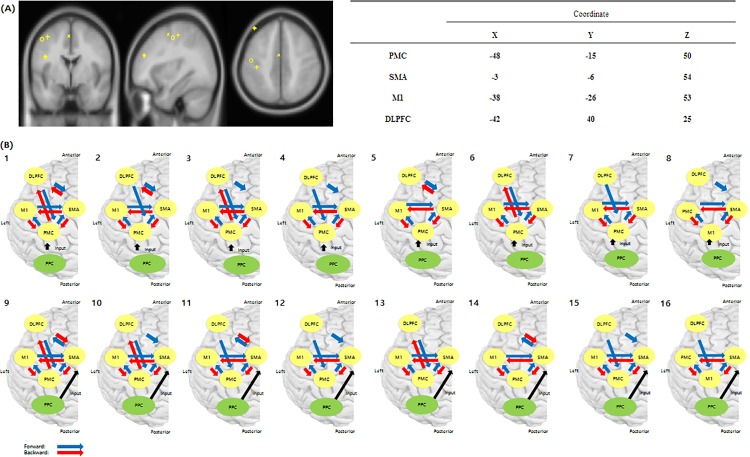
Regions of interest (ROIs) and connectivity models constructed by anatomical and structural imaging and computational modeling. (A) The regions of interest (ROIs) were PMC, SMA, DLPFC, and M1. (B) Connectivity models were constructed from anatomical and structural imaging and computational modeling. An extrinsic input through the posterior parietal cortex (PPC) entered the PMC or SMA, which was connected to M1, SMA, and DLPFC. PMC (+), Premotor Cortex; SMA (X), Supplementary Motor Area; M1 (O), Primary Motor Cortex; DLPFC (◈), Dorsolateral Prefrontal Cortex.

The regions of interest (ROIs) were set as M1, SMA, PMC, and DLPFC (Fig 2). We restricted ROIs to the left hemisphere because all the subjects who participated in our study were right handed. We employed the coordinates reported by Hanakawa et al., as prior source location means, because this experiment used a task paradigm and included the relevant ROIs for the chosen task [30].

### DCM specification

We tested whether the propagation of neuronal activity in the DLPFC affected primary and secondary motor areas using EEG data. On the basis of anatomical studies [44], a structural image suggesting the presence of such connections in humans [30], and a computational model of cognitive and motor control [44], we constructed the following DCM structures: An extrinsic input through the PPC entering the PMC and connecting to M1, SMA, and DLPFC and another extrinsic input through the PPC into the SMA and connecting to M1, PMC, and DLPFC [45, 46]. Input from primary visual cortex to the PPC through visual stimulation is a common pathway for both ME and MI, and the next connection is either from the PPC to the PMC or from the PPC to the SMA. We choose input from either the PMC or SMA. We reduced the nodes of the region and hypothesis model in order to perform exact model selection through the Bayesian theorem based on prior knowledge, because a larger number of nodes resulted in poor Bayesian model selection. The DLPFC was connected to the SMA and PMC. Condition-specific modulation of coupling due to external input did not influence all intrinsic coupling connections between regions [47, 48]. Based on intrinsic coupling between regions, we set up 16 models of connectivity representing biologically plausible hypotheses regarding changes in interregional coupling among ROIs during the performance of the right finger-tapping task. As the task was visually triggered, although PPC-SMA or PPC-PMC connectivity was supposed to be directly driven by task-dependent influences, the PPC was not included in the connection matrix because, with a large number of nodes in the region, Bayesian model selection based on the Bayesian theorem was poor. We investigated connections between the DLPFC and primary and secondary motor areas related to cognition and motor activity. According to setting up an interregional coupling for plausible hypotheses, we constructed 16 models according to exclusion of connections from the DLPFC to M1 and M1-DLPFC (Fig 2) because interregional couplings between the DLPFC and M1 and M1 and the DLPFC were functionally vague connections [48].

### Bayesian model selection (BMS)

We applied Bayesian model selection (BMS) using Bayesian Model Averaging with family-level inference and fixed-effects analysis (FFX) to determine the most likely model given the data [44]. First, we identified the region of input. To identify the type of input taking family-level inference into account, we organized models into families related to the PMC or SMA. A partition F that divided S into k~1:K disjoint subsets was constituted. Family k, which contained all models, was incorporated by subset and the k-th subset contained Nk models. We paid particular attention to avoid any unwanted bias in our inferences in order to establish a uniform prior at the family level [49]:

$$\rho (\int k)=\frac{1}{K}$$ (1)

if the equation looked upon this as model level [50]

$$\rho (\int k)=\sum_{m\epsilon \int k}\rho (m),$$ (2)

the value created applied to the uniform family prior [26]

$$\rho (m)=\frac{1}{KN_{k}}\forall m\in \int k$$ (3)

the relevant posterior model probabilities added up, and then resulted in the posterior distribution over families. [51]

$$\rho (\int k|Y)=\sum_{m\epsilon \int k}\rho (m|Y)$$ (4)

We divided the two partitions of family level into input regions: the PMC and SMA. Because the input most likely entered the dominant region, our study depended on the dominant result of family-level inference regarding the modulatory structure. Then, we assigned each of the models in the dominant family and analyzed BMS using FFX. These processes were performed on correct ME responses, correct MI responses, incorrect ME responses, and incorrect MI responses.

### Statistical analysis

Statistical analysis was performed using SPSS v20. One-way analysis of variance (ANOVA) was used to determine whether the selected model using FFX had a significant probability among the ROIs. For this analysis, we compared subjects and ROIs using the selected model. The difference between coupling parameters of each response (i.e., between correct ME and MI responses) was analyzed using Student’s *t*-tests and alpha slippage was corrected for multiple comparisons using the false discovery rate (FDR) method.

## Results

**Fig 3:**
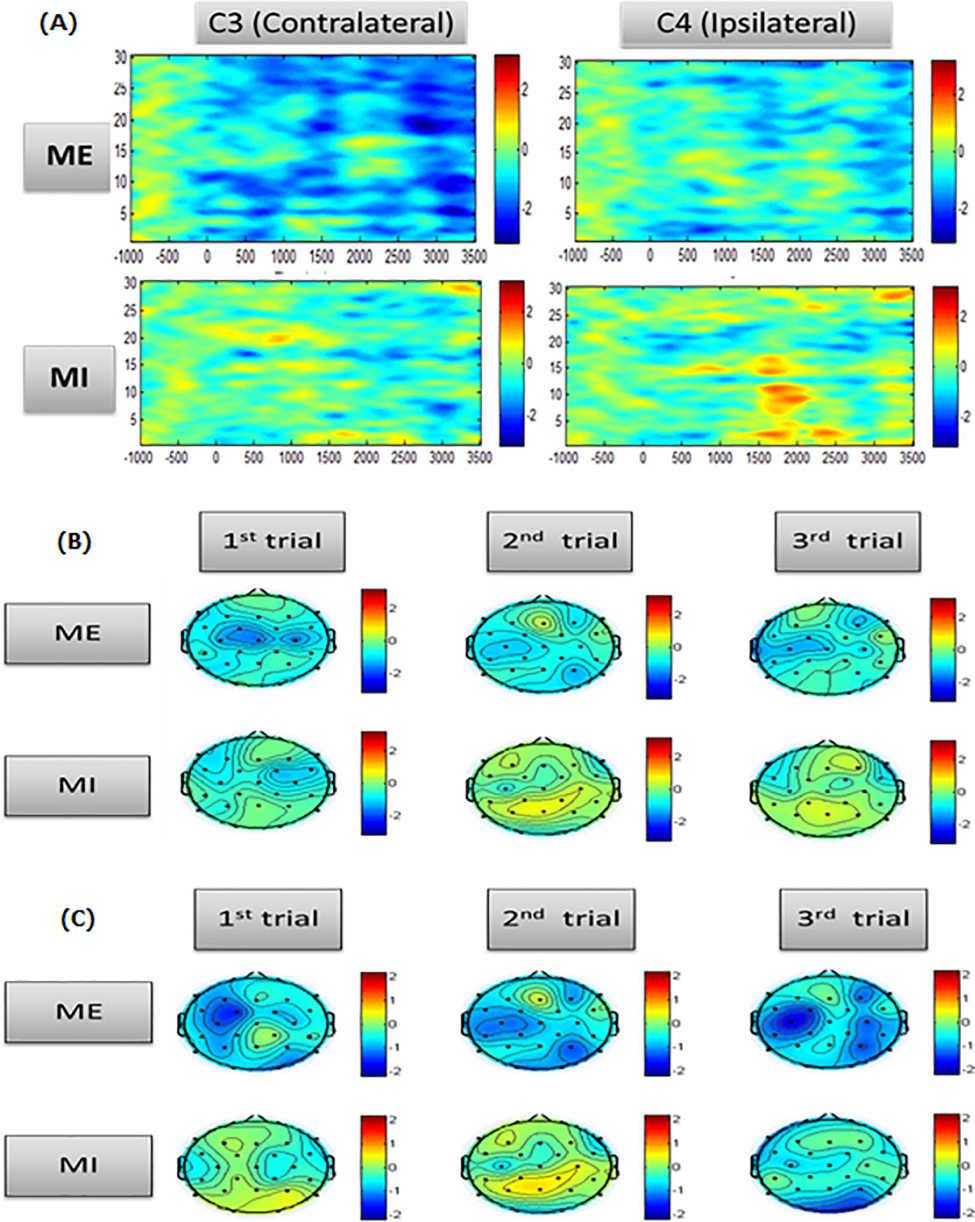
Event-related spectral perturbation over the C3 and C4 electrodes and topography for the mu and beta bands in each trial. (A) Event-related spectral perturbation over the C3 and C4 electrodes during motor execution (ME) and motor imagery (MI), (B) Topography at the mu band in each trial, (C) Topography at beta bands in each trial.

**Fig 4:**
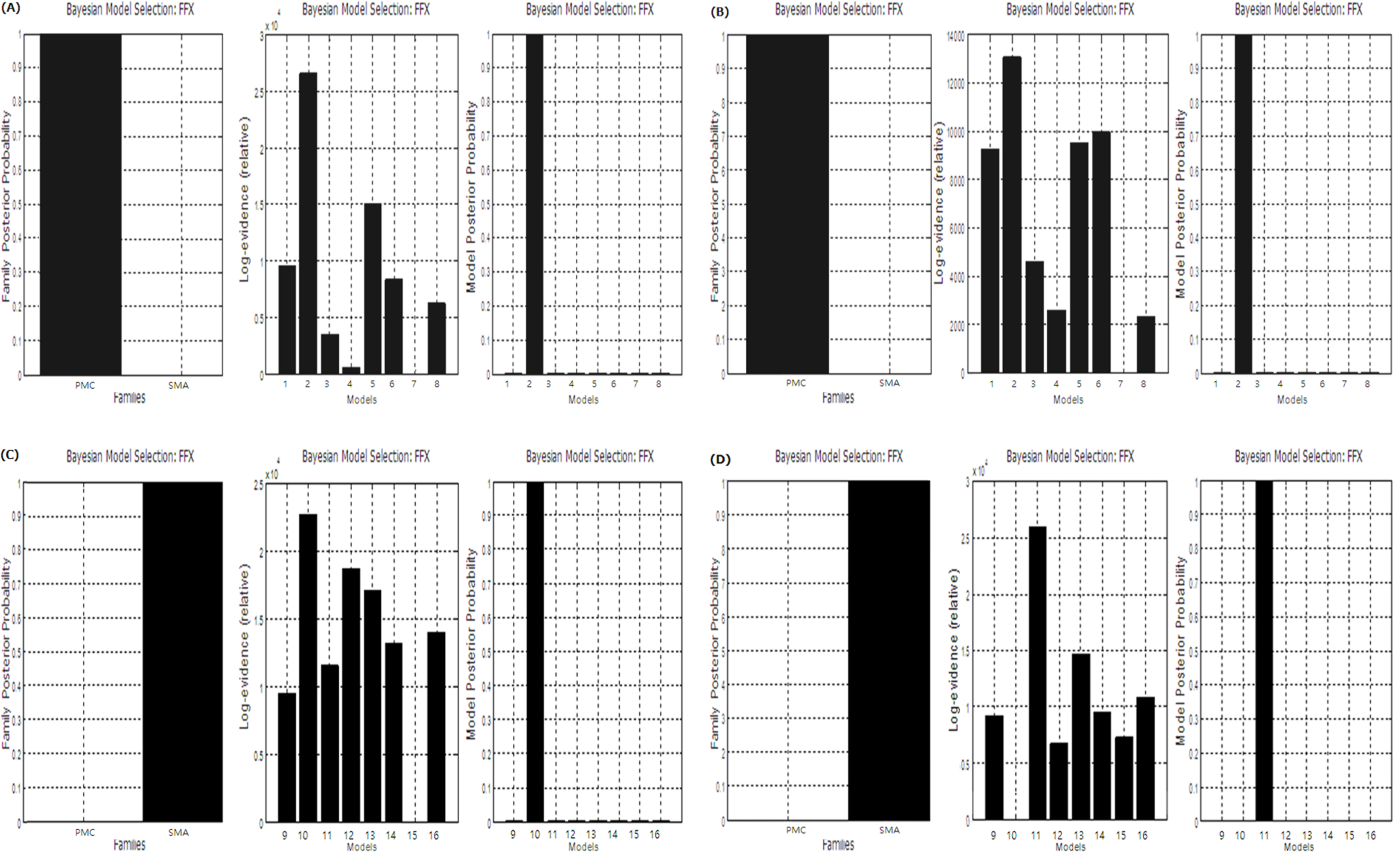
Family-level analysis and Bayesian model selection (BMS). (A) Correct responses during motor execution (ME), (B) Incorrect response during ME, (C) Correct responses during motor imagery (MI), and (D) Incorrect responses during MI.

Subjects performed 20 MI and 20 ME blocks on the same day with the task order randomized between subjects. The accuracy of a task was based on the numbers of correct and incorrect responses. Some subjects performed perfectly, responding to all tasks as intended. In contrast, other subjects performed either an exact button press or an inexact button press. The 20 blocks included both correct and incorrect responses. We collected the responses for all blocks in all subjects and then divided them into correct and incorrect groups. The total correct task performance of ME was 90.4%, while that of MI was 84.5%. We observed that 11 of 20 subjects performed 20 of 20 correct responses during ME, and 9 of 20 subjects performed 20 of 20 correct responses during MI. The average MIQ-RS score of the participants was 13.03±1.07, the kinesthetic MIQ-RS was 6.7±0.53, and the visual imagery MIQ-RS was 6.5±0.53 (Table 1). A two-sample t-test indicated no significant difference between ME and MI performance. As mentioned in the EEG Recording and Data Processing section, the ERSP analysis was performed as a preprocessing procedure when the EEG signals were preprocessed with EEGLAB. Results of the ERSP analysis showed ERD appeared at C3 in the mu band during ME. For the mu band at C4, an ERD pattern also appeared, but was less significant than that at C3. Although a similar phenomenon was found during MI, the ERD pattern at C3 was less significant in the mu band, as well as at C4 (Fig 3A). Spectral power analysis showed when the mu band was targeted during ME, a distinct result in the sensorimotor area was seen, but MI was associated with an ambiguous ERD pattern. In the beta band, ERD was observed over the sensorimotor area during ME and became more distinct during the 3rd trial than the 1st trial (Fig 3B). MI elicited a vague ERD pattern in the beta band, which also became relatively distinct by the 3rd trial (Fig 3C). We used family-level inference to determine the dominant region of input divided into the PMC and SMA. As a result, ME, including correct and incorrect responses, was associated with the PMC as its input region (Fig 4A and 4B). As previously mentioned, we restricted the set models to the predominant input. Accordingly, we analyzed only PMC models 1–8 using BMS by FFX inference. Consequently, model 2, representing the connected DCM-B matrix, showed the best fit across the correct PMC ME responses (Fig 4A). Moreover, when we analyzed only PMC family models for incorrect ME responses, model 2 provided the best fit (Fig 4B). Conversely, MI including correct and incorrect responses was associated with the SMA as the region of input. Results from family-level inference by FFX showed that the family of models with SMA as the input to the network had a better fit than the family of models with PMC as the input (Fig 4C and 4D). When we performed family-level inference, model 2 with the SMA input as model 10 on the BMS graph had a better fit than the other models and produced correct MI responses (Fig 4C). Moreover, we demonstrated that model 3 with the SMA input as model 11 provided the best fit, according to the same procedure used for incorrect MI responses (Fig 4D).

**Table 1:** Participant MIQ-RS score.

	Mean (SD)
**Total MIQ-RS**	13.03 (1.07)
**Kinesthetic Imagery**	6.7 (0.53)
**Visual Imagery**	6.5 (0.53)

MIQ-RS: Movement Imagery Questionnaire-Revised second version; SD: standard deviation

**Fig 5:**
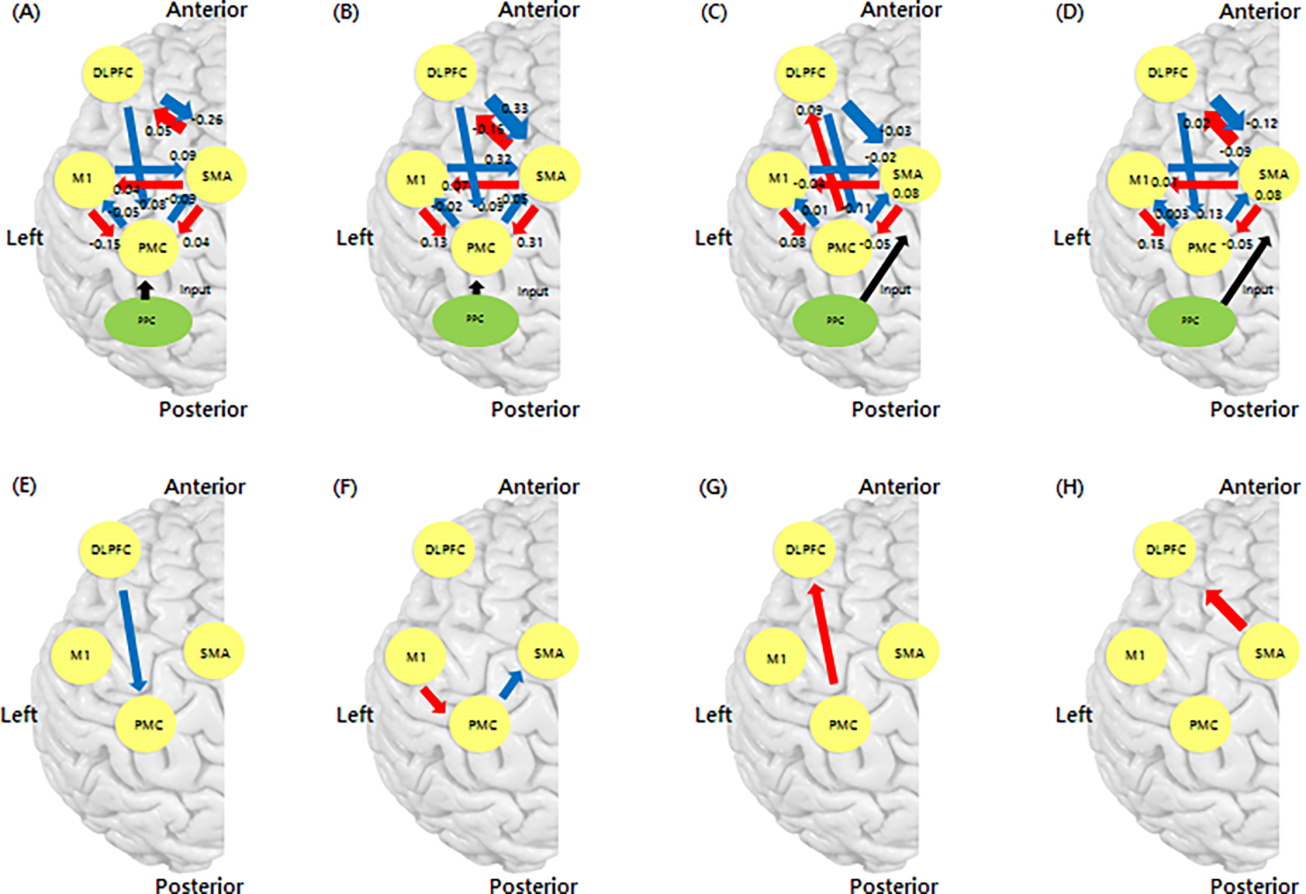
DCM coupling strength based on modulatory connectivity (DCM-B matrix). (A) Correct responses during motor execution (ME), (B) Incorrect response during ME, (C) Correct responses during motor imagery (MI), (D) Incorrect responses during MI, (E) Higher coupling strength during ME compared with correct MI, (F) Higher coupling strength for MI compared with correct ME, (G) Connectivity characteristic of correct MI responses compared with incorrect MI, and (H) Connectivity characteristics of incorrect MI responses compared with correct MI.

We performed statistical analysis using one-way ANOVA when comparing subjects and ROIs in model 2 for correct ME and MI responses. We demonstrated that model 2 with both correct and incorrect ME responses exhibited significant forward and backward interactions between nodes (p<0.05; Fig 5A and 5B) (Table 2). Model 10 with correct MI responses also showed significant interactions between nodes. Additionally, a significant interaction was observed in model 11 with incorrect MI responses (p<0.05; Fig 5C and 5D) (Table 2). We excluded PMC-DLPFC and SMA-DLPFC when performing the Student’s t-test because model 2 in correct ME responses and model 11 in correct MI responses did not have the same coupling parameters. A significant difference was identified in the DLPFC-PMC coupling parameter for correct ME responses compared with coupling parameters in correct MI responses using a Student’s *t*-test corrected for FDR (p<0.05; Fig 5E). The DLPFC-PMC coupling parameter (mean coupling parameter: 0.08, standard deviation: 0.28) was a forward network. In contrast, for correct MI responses, PMC-SMA and M1-PMC coupling parameters differed significantly when compared with coupling parameters during correct ME responses using a Student’s *t*-test corrected for FDR (p<0.05; Fig 5F). PMC-SMA (mean parameter: 0.08, standard deviation: 0.32) was a forward coupling parameter and differed in connectivity value from PMC to SMA and this connectivity mediated more than correct ME responses during correct MI responses. Despite the behavioral differences between correct and incorrect responses in ME, both conditions possessed similar network connectivity characteristics. However, correct and incorrect MI responses possessed different network connectivity characteristics. Correct MI responses were associated with connectivity from the PMC to the DLPFC (Fig 5G), while incorrect MI responses were associated with comparable connectivity from the SMA to the DLPFC when compared to correct MI responses, there was no statistical significance in connectivity because of the difference between the groups in terms of SMA to DLPFC effective connectivity (Fig 5H).

**Table 2:** Coupling parameters from dynamic causal modeling.

ROI			PMC	SMA	M1	DLPFC
**PMC**	**ME**	**Correct**	0	0.04 (0.23)	-0.15 (0.21)	0.08 (0.28)*
		**Incorrect**	0	0.31 (0.65)	0.13 (0.31)	-0.09 (0.17)
	**MI**	**Correct**	0	-0.05 (0.17)	0.08 (0.38)*	-0.11 (0.23)
		**Incorrect**	0	-0.05 (0.15)	0.15 (0.29)	0.13 (0.26)
**SMA**	**ME**	**Correct**	-0.09 (0.15)	0	0.09 (0.27)	-0.26 (0.5)
		**Incorrect**	-0.05 (0.15)	0	0.32 (0.59)	0.33 (0.46)
	**MI**	**Correct**	0.08 (0.32)*	0	-0.02 (0.14)	-0.03 (0.13)
		**Incorrect**	0.08 (0.3)	0	-0.09 (0.18)	-0.12 (0.2)
**M1**	**ME**	**Correct**	-0.05 (0.13)	0.04 (0.27)	0	0
		**Incorrect**	-0.02 (0.03)	0.07 (0.07)	0	0
	**MI**	**Correct**	0.01 (0.19)	-0.04 (0.13)	0	0
		**Incorrect**	0.003 (0.09)	0.01 (0.05)	0	0
**DLPFC**	**ME**	**Correct**	0	0.05 (0.35)	0	0
		**Incorrect**	0	-0.16 (0.35)	0	0
	**MI**	**Correct**	0.09 (0.2)	0	0	0
		**Incorrect**	0	0.02 (0.06)	0	0

* Asterisk mark indicates a significant result (p<0.05).

Mean (standard deviation, SD); DLPFC, dorsolateral prefrontal cortex; ME, motor execution; MI, motor imagery; M1, primary motor cortex; PMC, premotor cortex; ROI, region of interest; SMA, supplementary motor area.

## Discussion

We proposed that the DLPFC is part of a network for cognitive and motor activities during ME and MI based on a past effective connectivity study. As mentioned in the DCM Specification in the Methods section, we supposed that ME and MI responses received common stimulation from the PPC and then sent that information to the PMC and SMA, respectively. In a previous primate study, the parieto-dependent motor area received information from the PPC that was involved in a series of sensory-motor transformations and connected mainly to area F5. Area F5 plays a role in the execution of behaviors that include grasping, manipulation, and hand movements and the F5 area corresponds to Brodmann area 6, including the premotor cortex [48]. Therefore, connection from the parieto-dependent motor area to the PPC suggests a connection between the PPC and PMC in the case of human-beings. Other previous primate studies showed that the posterior parietal lobe sent information to area F6 [13, 52]. The suggested role for area F6 corresponding to the mesial part of human 6aß (SMA) is that its neurons constitute a system that controls potential actions encoded in the lateral parieto-frontal circuits. Normally, even when the neurons encoding these potential actions are activated, movement is not initiated. The control system represented by area F6 causes the initiation of movement when external contingencies and motivational factors allow [50]. Thus, hypothetically, the PMC played a role in actual motor action in the presence of external stimulation during correct and incorrect ME responses; however, the SMA might play a role in the preparation of behavior because of inhibition of execution during correct and incorrect MI responses. For these reasons, the input received originated from different regions depending on whether the task was ME or MI.

Correct ME and MI responses had effective connectivity nodes shared by the DLPFC-SMA and the DLPFC-PMC (Fig 5A and 5B). This result agrees with a previous primate study [50]. Previous studies [48, 50] indicated that the motor system was prefronto-dependent corresponding to the connection between motor areas and prefrontal and parieto-dependent cortical motor areas that correspond to the connection between the parietal area and posterior motor areas in the human. Higher-order cognitive information associated with long-term motor plans and motivation was sent to the prefronto-dependent motor areas, while the parieto-dependent motor areas received sensory information derived from the parietal lobe and used it for action. The prefronto-dependent motor areas received their main cortical connections from the prefrontal cortex, area F6 and area F7, implying pre-SMA and dorsal PMC, respectively [50]. In addition, although the input areas were different for correct and incorrect ME responses and incorrect MI responses, the connectivity patterns were similar.

Research comparing ME and MI using fMRI showed that similar brain areas were activated during movement and imagery tasks, including the PMC, SMA, and DLPFC [28, 49]. A previous study showed M1 was more activated during execution than imagination upon time course analysis. Thus, although M1 produced marked activity during the movement task in that previous study, minimal activity was observed during the imagery task; these connectivity results during ME and MI agree with the present results. To compare certain differences between identical coupling parameters, we analyzed the two responses using Student’s t-tests. As a result, the two responses showed significantly different coupling parameters for DLPFC-PMC, PMC-SMA and M1-PMC (Fig 5E and 5F). A previous primate lesion study showed that area F7 neurons originating in the DLPFC were associated with performance of goal-directed movements in response to arbitrary external stimuli [52]. Regarding DLPFC-PMC connectivity (Fig 5E), a study focusing on attention to action suggested that effective connectivity between the DLPFC and PMC was enhanced by attention to action compared with attended performance of a simple motor task and unattended performance of the same movements [53]. As mentioned in the Results section, forward network for the DLPFC-PMC coupling parameter (mean coupling parameter: 0.08, standard deviation: 0.28) meant that connections from the DLPFC to the PMC might modulate more than correct MI responses when finger tapping with the right hand. In summary, DLPFC-PMC connectivity during movement execution is possibly involved in attention to action than during imagining movement. In addition, motor control to perform goal-directed movements was higher during ME than MI because pressing a button required goal-directed movement. This suggests that DLPFC-PMC coupled connectivity plays a role in the motor control needed to perform goal-directed movement [54, 55].

As mentioned above, correct ME and MI responses both involved PMC-SMA and M1-PMC coupling. These findings imply that these areas play major roles in conscious movement intention and movement awareness [46]. These network strengths were stronger for correct MI responses than correct ME responses (Fig 5F). Imagined movement could represent the result of conscious access to the content of the intention, which would reflect image expression in the mind [46]. Therefore, the coupling strength of correct MI responses including PMC-, SMA-, and M1-related motor intention was higher than for ME responses, possibly because young healthy subjects were used to perform MI with higher subjective feelings of conscious intention and movement awareness than when performing ME. We might assume that, compared with the reference MIQ-RS score from a previous study [35], the present scores indicate that subjects adequately performed the imagery task.

There were differences in receiving information from the DLPFC between correct and incorrect MI responses (Fig 5G and 5H). SMA-DLPFC coupling was observed during incorrect MI responses, and the presence of connectivity from PMC to DLPFC was observed during correct MI responses. SMA-DLPFC coupling has been shown to play a role in the motor control needed to move a finger [3]. Additionally, PMC-DLPFC coupling is involved in preparation for movement [2]. This finding suggests information is sent from the PMC to the DLPFC when subjects executed imagined movements during MI and reflects a consistent imagery intention before imagined movement.

Overall, this study’s results indicated that DLPFC-PMC and DLPFC-SMA connectivity were similar during ME and MI and based on information from a previous study, the motor system may recruit prefronto-dependent and parieto-dependent motor areas during ME and MI. These results showed that cognitive demand was required for the motor control needed to perform goal-directed movements during both the ME and MI tasks. Correct ME responses might have required more connectivity for achieving that action than did correct MI responses, because physically pressing the button evoked DLPFC-PMC coupling. Other common coupling nodes included PMC-SMA and M1-PMC. These coupled areas are important components of conscious movement intention and movement awareness. Therefore, the common characteristics of correct ME and MI responses required attention for the action, but correct ME responses were higher than those of MI when actually pressing the button. Second, correct ME and MI responses were associated with the PMC-SMA and M1-PMC coupling parameters because they required motor intention and motor awareness. These network strengths might have been stronger for correct MI responses because the young healthy subjects performed MI with higher subjective feelings of conscious intention and movement awareness than for ME, which was reflected in the MIQ-RS scores.

This study has some limitations. First, our sample size was insufficient to analyze the difference between the correct response and incorrect response models. Although the correct response model revealed effective connectivity between motor and cognitive processes during ME and MI, the incorrect response model was inadequate to permit statistical analysis. In addition, we could not perform statistical analysis in order to differentiate between the correct and incorrect response models because this study could not have any more data to make the estimation using the BEM more accurate. Therefore, further research is needed with a large number of subjects and task blocks to investigate the relationship between motor behavior and cognition during ME and MI. Second, the ROIs were insufficient to examine the networks of interest within the whole brain related to motor behavior and cognition during ME and MI, even though we decided to decrease the ROIs based on Bayes theorem in order to approximate exact model selection. Third, we could not calculate subject-specific head models although we used a template head model that included a cortical mesh of 8,196 vertices provided by SPM. In addition, we could not offer a number of channels greater than the channels we measured, thus even though we used BEM for correction in order to solve some the forward problem, the BEM had the drawback of being computationally complex. Therefore, further research is needed to calculate subject-specific head models and the optimum number of channels. Fourth, we did not consider connectivity from the PPC to the PMC and the PPC to SMA. Although there are two reasons we did not consider this model, including connections from the PPC to PMC and PPC to SMA and the accuracy of Bayesian model computation and restriction of brain areas to only motor and cognition areas, we maintain the connectivity between motor areas and the posterior parietal cortex should have been considered. Therefore, further research is needed to analyze a connectivity model that includes the PPC and PMC model and the PPC and SMA. Fifth, we also could not consider ROIs in the left and right hemisphere. We considered restricting ROIs to the left hemisphere because all subjects were right handed. However, we did not contemplate the connection between the right and left hemisphere. Therefore, further research is needed to map the right and left hemisphere connection. Sixth, we could not use individual scans such as fMRI images. Although we used the template head model and BEM method, EEG positions were, of necessity, inaccurate relative to underlying brain structure by individual. Therefore, further research is needed to acquire individual source reconstruction information. Lastly, we could not consider the interaction of timing with movement including the occurrence of dynamics during finger tapping between trials in the DCM analysis. Therefore, further research is needed to analyze the interaction between timing and movement dynamics between trials.

## Conclusion

We showed evidence for effective connectivity between motor and cognitive processes during ME and MI. First, when the executed movement resulted in either correct or incorrect responses, the PMC received input from the PPC; however, the SMA received input from the PPC during the MI task. Moreover, our study showed effective connectivity between the DLPFC and secondary motor areas as well as connectivity between primary and secondary motor areas during both ME and MI tasks, in accord with previous primate and brain activation studies. We found that the DLPFC-PMC were more coupled during correct ME than correct MI responses, and connectivity between the PMC-SMA and the M1-PMC was stronger during correct MI compared to correct ME responses. These results indicate that effective connectivity is different during executed and imagined movement, so there might be different processes between ME and MI. Third, although there was a behavioral difference between correct and incorrect responses during ME and MI, correct and incorrect responses during MI were associated with different network connectivity characteristics.

## Supporting information

S1 Table Vector of summed log evidences of motor execution (ME) for Bayesian model selection (BMS). (DOCX)

S2 Table Vector of summed log evidences of motor imagery (MI) for Bayesian model selection (BMS). (DOCX)
