# Supplementary material for: Changes in network connectivity during motor imagery and execution
Source: PLoS One. 2018 Jan 11;13(1):e0190715. doi: 10.1371/journal.pone.0190715 (PMC5764263; doi:10.1371/journal.pone.0190715)
Supplement: S1 Table — (DOCX) [file pone.0190715.s001.docx]

**Supporting Data**

**S1 Table. Vector of summed log evidences of motor execution (ME) for Bayesian Model selection (BMS)**

| **Model** | **Motor Execution (ME)** | |
| --- | --- | --- |
|  | **Correct** | **Incorrect** |
| 1 | 2.155081856873638e+05 | 8.317104541691906e+04 |
| 2 | 2.326504434050680e+05 | 8.694832475596634e+04 |
| 3 | 2.094771755757656e+05 | 7.846084660133191e+04 |
| 4 | 2.065871430703066e+05 | 7.646876952082806e+04 |
| 5 | 2.210332877177193e+05 | 8.336024760107936e+04 |
| 6 | 2.143576694346067e+05 | 8.382128760304734e+04 |
| 7 | 2.059789855829025e+05 | 7.387560650415969e+04 |
| 8 | 2.122146279126032e+05 | 7.620399106858915e+04 |
