# Supplementary material for: Changes in network connectivity during motor imagery and execution
Source: PLoS One. 2018 Jan 11;13(1):e0190715. doi: 10.1371/journal.pone.0190715 (PMC5764263; doi:10.1371/journal.pone.0190715)
Supplement: S2 Table — (DOCX) [file pone.0190715.s002.docx]

**Supporting Data**

**S2 Table. Vector of summed log evidences of motor imagery (MI) for Bayesian Model selection (BMS)**

| **Value of Model** | **Motor Imagery (MI)** | |
| --- | --- | --- |
|  | **Correct** | **Incorrect** |
| 9 | 2.216537433079954e+05 | 1.208848039760026e+05 |
| 10 | 2.359557092180738e+05 | 1.116779568945040e+05 |
| 11 | 2.164044721554270e+05 | 1.377416256196867e+05 |
| 12 | 2.276894013582471e+05 | 1.184294071750515e+05 |
| 13 | 2.255530752509791e+05 | 1.263249805578152e+05 |
| 14 | 2.271027028587433e+05 | 1.212700888834289e+05 |
| 15 | 2.167601979926631e+05 | 1.189849451795160e+05 |
| 16 | 2.226607469576567e+05 | 1.224932603160980e+05 |
